# Fortification of tropical macroalgae (*Gelidium* sp., *Gracilaria* sp., *Sargassum* sp., *Ulva* sp.) enhances mineral profile, strength, and ruminal degradability of mineral blocks for beef cattle

**DOI:** 10.5455/javar.2026.m1012

**Published:** 2026-03-09

**Authors:** Gunawan Gunawan, Ahmad Sofyan, Erna Winarti, Harwi Kusnadi, Awistaros Angger Sakti, I Nyoman Guna Darma, Ririen Indriawaty Altandjung, Wulandari Wulandari, Heru Ponco Wardono, Zein Ahmad Baihaqi, Dwi Eny Djoko Setyono, Sutardi Sutardi, Karen Jean Harper

**Affiliations:** 1 Research Center for Animal Husbandry, National Research and Innovation Agency, Bogor 16911, Indonesia; 2 Graduate School of Faculty of Animal Science, Bogor Agricultural University (IPB University), Dramaga-Bogor 16680, Indonesia; 3 Research Center for Food Technology and Processing, National Research and Innovation Agency, D.I. Yogyakarta 55861, Indonesia; 4 Research Center for Food Crops, National Research and Innovation Agency, Bogor 16911, Indonesia; 5 School of Health, Medical and Applied Sciences, Central Queensland University, Rockhampton, Qld 4702, Australia

**Keywords:** Cattle, macroalgae, mineral blocks, ruminal degradability

## Abstract

**Objectives:** Mineral blocks are widely used for ruminant supplementation, but their low mineral availability and weak physical properties often limit nutrient utilization. This study evaluated the effects of fortifying mineral blocks with tropical macroalgae on their mineral composition, physical quality, ruminal degradability, and palatability in Ongole crossbred bulls.

**Materials and Methods:** The study employed a completely randomized design (CRD) with five treatments, comprising one non-fortified control (MBNF) and four macroalgae-fortified mineral blocks (MBFM) containing *Gelidium* sp., *Gracilaria* sp., *Sargassum* sp., or *Ulva* sp., each replicated three times. The mineral composition was determined by X-ray fluorescence (XRF), and the physical properties were evaluated in accordance with the Indonesian National Standard (SNI 1974:2011). *In vitro* ruminal degradation and gas production were measured over a 72-h incubation in five replicates per treatment, whereas palatability was assessed in 10 Ongole crossbred bulls. Data were analyzed using analysis of variance (ANOVA), and mean differences were tested by Duncan’s Multiple Range Test at *p* < 0.05.

**Results:** Fortification with *Gelidium* sp. significantly increased Potassium (K), Iron (Fe), Manganese (Mn), and Copper (Cu) contents compared with MBNF (*p* < 0.05). Compressive strength improved by up to 69%, particularly in *Gelidium*-fortified blocks (*p* < 0.05). Ruminal degradability was enhanced, with dry matter digestibility and organic matter digestibility increasing by 39% and 36%, respectively (*p* < 0.05). Palatability tests showed no significant differences in block consumption between MBFM and MBNF (*p* > 0.05).

**Conclusions:** Fortifying mineral blocks with *Gelidium* sp. improved mineral enrichment, compressive strength, and ruminal degradability without reducing palatability, indicating its potential as an effective mineral supplementation strategy for Ongole crossbred bulls.

## 1. Introduction

Smallholder cattle production in Indonesia relies on cut-and-carry feeding systems, where farmers supply limited amounts of forage and agricultural by-products [1]. Feed availability and quality fluctuate seasonally, particularly during the dry period, which often exacerbates nutrient and mineral imbalances. This practice often results in inadequate or imbalanced mineral intake because it is difficult to accurately assess livestock requirements. Such limitations lead to mineral deficiencies that reduce feed efficiency, growth rate, reproductive performance, and overall profitability. Mineral deficiencies disrupt metabolic processes, compromise immune function, and impair growth and reproduction, ultimately reducing productivity [2, 3].

Mineral supplementation through mineral blocks is a practical solution for smallholder cattle systems, enabling animals to self-regulate their intake and improve overall nutrient supply [2, 4]. However, conventional mineral blocks may have suboptimal mineral content and bioavailability, and their physical durability can be insufficient under field conditions.

Macroalgae offer a promising fortification strategy, as they are rich in essential and trace minerals, such as Calcium (Ca), Sodium (Na), potassium (K), and manganese (Mn), accounting for up to 97% of their mineral profile [5]. Additionally, their polysaccharides (e.g., alginates) serve as natural gelling and binding agents, enhancing compressive strength, while their bioactive compounds may improve palatability and digestibility [6, 7, 8, 9].

Research on macroalgae-fortified mineral blocks in Indonesian smallholder systems is still limited. This study aimed to evaluate the effects of fortifying mineral blocks with four tropical macroalgae species (*Gelidium* sp., *Gracilaria* sp., *Sargassum* sp., and *Ulva* sp.) on mineral content, physical characteristics, ruminal degradability, and palatability in Ongole crossbred bulls, providing insights into locally relevant supplementation strategies.

## 2. Materials and Methods

### 2.1. Ethical approval

The research experiment was approved by the Ethics Committee on Animal Care and Use of the National Research and Innovation Agency, Indonesia, with the number 191/KE.02/SK/10/2023.

### 2.2. Collecting samples and location

Fresh tropical macroalgae were collected from the Sepanjang Beach area in Kemadang Village, Tanjungsari Sub-district, Gunungkidul Regency, Special Region of Yogyakarta, Indonesia, at the coordinates 8°08’14.9” S and 110°34’07.9” E. The mineral block palatability assessment was conducted at the Andini Mulyo farmer group in Bleberan Village, Playen Sub-district, Gunungkidul Regency, Special Region of Yogyakarta, Indonesia, at the coordinates 7°57’29” S and 110°30’23” E.

### 2.3. Mineral block preparation

Fresh macroalgae were collected from Sepanjang Beach (8°08’14.9” S, 110°34’07.9” E), Kemadang Village, Tanjungsari Sub-district, Gunungkidul Regency, Yogyakarta, Indonesia. The biomass was washed under running freshwater, drained, and sun-dried for 2–3 consecutive sunny days at ambient temperatures of 30–33°C and relative humidity of 65–75%, until the moisture content was below 14%. The dried material was cut, ground using a hammer mill, and sieved through a 50-mesh screen (approximately 0.3 mm particle size) to obtain a uniform powder. The resulting macroalgae meal was stored in airtight containers at room temperature (25–30°C) until used to fortify mineral blocks.

Mineral blocks were fortified with 2% dry matter (DM) of four different types of macroalgae and compared with a non-fortified mineral block (MBNF). The four types of macroalgae utilized were *Gelidium* sp. (MBFM-GE), *Gracilaria* sp. (MBFM-GR), *Sargassum* sp. (MBFM-SA), and *Ulva* sp. (MBFM-UL). The formulation of the MBFM (MBFM-GE, MBFM-GR, MBFM-SA, MBFM-UL) and MBNF blocks is presented in Table 1.

**Table 1.** Composition of mineral block fortified by macroalga and control (non-fortified) in dry matter.

Composition	MBFM				MBNF
	*Gelidium* sp.	*Gracilaria* sp.	*Sargassum* sp.	*Ulva* sp.	
Salt (%)	38	38	38	38	40
Tri calcium silicate (%)	31	31	31	31	31
Mineral powder (%)	20	20	20	20	20
Peanut husk (%)	9	9	9	9	9
Macroalgae (%)	2	2	2	2	0
Total (%)	100	100	100	100	100

MBFM = mineral block fortified by macroalga; MBNF = mineral block non-fortified; salt = iodine salt, minimum NaCl content 94% as dry matter basis, in powder (size 50 mesh); mineral powder = mineral commercial with content of calcium 50%, potassium 25%, phosphorus 15%, magnesium 6%, sodium chloride 5%; peanut husk and macroalgae in powder (size 50 mesh).

The ingredients of each block were mixed evenly, and 150–175 ml of water per kilogram was added. Then, the mixture was stirred until a dough-like consistency was achieved. The dough was packed and compacted into a polyvinyl chloride pipe measuring 8.5 cm in diameter and 12 cm in height. Afterward, the dough was removed from the pipe, producing a firm, wet mineral block. Wet mineral blocks were then dried at 26–30°C for 4–7 days.

### 2.4. Mineral content assessment

Samples of all mineral blocks were analyzed using an X-ray fluorescence (XRF) analyzer, following the Omnian Standard method with Helium, to determine the mineral composition of MBNF and MBFM from four species of macroalgae. The concentrations of Ca, phosphorus (P), K, Na, sulfur (S), Cu, Fe, Mn, and zinc (Zn) were measured in 3 replicates.

### 2.5. Physical properties assessment

Samples of mineral blocks MBFM and MBNF were measured and tested for physical properties, including height (cm), diameter (cm), volume (cm^3^), weight (kg), specific gravity (kg/dm^3^), and compressive strength (kg/cm^2^). The mineral block compressive strength test followed the Indonesian National Standard method (SNI 1974:2011) for testing the compressive strength of cylindrical concrete specimens. Each mineral block was tested with 5 replicates.

### 2.6. In vitro ruminal degradation assessment

The *in vitro* ruminal degradation of the basal *Pennisetum purpureum* diet, supplemented with different mineral blocks, was assessed using the gas production method [10]. Samples of 300 mg (dry matter basis) for each treatment were incubated in a mixture of rumen fluid and buffer in 100 ml-capacity serum bottles (Duran^®^, Germany). The treatments in the *in vitro* test included a basal diet + MBNF, a basal diet + MBFM-GE, a basal diet + MBFM-GR, a basal diet + MBFM-SA, and a basal diet + MBFM-UL (Table 2). Each treatment was tested using 5 replicates.

**Table 2.** Treatment in the *in vitro* test of mineral block fortification.

Treatments	Composition of feed
MBNF	A basal diet + MBNF
MBFM-GE	A basal diet + MBFM-GE
MBFM-GR	A basal diet + MBFM-GR
MBFM-SA	A basal diet + MBFM-SA
MBFM-UL	A basal diet + MBFM-UL

A basal diet used *Pennisetum purpureum*; MBNF = mineral block non-fortified; MBFM-GE = mineral blocks fortified by *Gelidium* sp.; MBFM-GR = mineral blocks fortified by *Gracilaria* sp.; MBFM-SA = mineral blocks fortified by *Sargassum* sp.; MBFM-UL = mineral blocks fortified by *Ulva* sp.

Gas production was observed at incubation times of 2, 4, 6, 8, 12, 24, 36, 48, and 72 h. The prediction of the gas production function over time units was carried out using the equation proposed by Ørskov and McDonald [11]:


\[
P = a + b(1-{e^{-ct}})
\]

With: *P* = total gas production (ml),

*a* = gas production from soluble fraction (ml),

*b* = gas production from the potential soluble part (ml),

*c* = gas production rate (ml/h),

*e* = Euler’s number.

The dry matter (DM) and organic matter (OM) of the substrate from each syringe were measured according to AOAC [12]. The percentage differences in DM and OM between the initial and post-incubation measurements, corrected with blank, were calculated as *in vitro* digestibility for 72 h using the following formula:


\[
\begin{array}{*{20}{l}}
{\rm IVDMD} = \frac{{\left[{{\rm DM}f-\, \left({{\rm DM}r-{\rm DM}b}\right)}\right]}}{{{\rm DM}f}}\;\times\;100\%\\
{\rm IVOMD} = \frac{{\left[{{\rm OM}f-\, \left({{\rm OM}r-{\rm OM}b}\right)}\right]}}{{{\rm OM}f}}\;{\times}\;100\%
\end{array}
\]

With: IVDMD = *In vitro* dry matter digestibility,

IVOMD = *In vitro* organic matter digestibility,

DM*f* = dry matter of feed,

DM*r* = dry matter of residue,

DM*b* = dry matter of blank,

OM*f* = organic matter of feed,

OM*r* = organic matter of residue,

OM*b* = organic matter of blank.

### 2.7. Mineral block palatability assessment

Ten Ongole crossbred bulls with an initial body weight (BW) of 220 ± 49.1 kg and aged between 12 and 18 months were placed in individual pens for the mineral block palatability assessment. Prior to the experiment, all bulls were treated with a macrocyclic lactone via subcutaneous ivermectin injection (1 ml/50 kg BW) to prevent parasitic infection.

**Figure 1.**
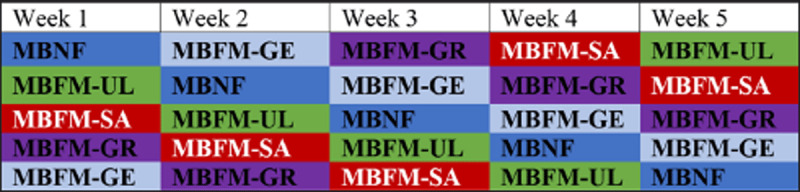
Weekly rotation of mineral blocks for each bull over 5 weeks during the feed palatability assessment. MBNF = mineral block non-fortified; MBFM-UL = mineral block fortified by *Ulva* sp.; MBFM-SA = mineral block fortified by *Sargassum* sp.; MBFM-GR = mineral block fortified by *Gracilaria* sp.; MBFM-GE = mineral block fortified by *Gelidium* sp.

The palatability of mineral blocks was assessed through free-choice feeding of Ongole bulls. Each bull had access to the five mineral blocks (MBNF, MBFM-GE, MBFM-GR, MBFM-SA, and MBFM-UL), which were hung at a height of 1.5 m from the pen floor, with a distance of 30 cm between each block. The mineral blocks were hung in the pen, arranged in a particular sequence, which was rearranged each week over a five-week period, ensuring that the bulls had the opportunity to consume each mineral block, and positioning did not skew results. The positioning of the five mineral blocks in the bull pen over the five-week period is shown in Figure 1. The block arrangement in each pen was rotated weekly and applied to all animals.

The bulls were offered forage at 10% BW as fed/day, wheat bran at 1% BW as fed/day, and access to mineral blocks *ad libitum*. The main forage provided was *Pennisetum purpureum*, while the supplementary forage included rice straw, corn stover, and peanut straw. The dry matter content of the forage mixture was 27.2%, and wheat bran was 89.2%. The forage and wheat bran were offered twice daily: 50% in the morning (approximately 06:00 to 07:00 h) and 50% in the afternoon (approximately 15:00 to 16:00 h). Fresh drinking water was provided *ad libitum* throughout the experimental period. The water troughs were refilled every morning and evening (approximately 15 L each time) to ensure the animals had access to drinking water at all times.

The parameters observed were bull BW and consumption of mineral block. The BW was measured to determine the required amount of forage and wheat bran to be fed. The body weight of bulls was recorded using a digital livestock scale (X-SAGO^®^, Indonesia; capacity 1.5 tons). Mineral blocks were weighed weekly using a digital bench scale (EK-5055^®^, Camry, China). Mineral block consumption per week was calculated by subtracting the current weight of the mineral block from the weight of the mineral block from the previous week.

### 2.8. Statistical analysis

This research employed a completely randomized design (CRD). The mathematical model used is as follows:


\[
{Y_{ij}} = \mu \; + \;{\alpha_i}\; + \;{\varepsilon_{ij}}
\]

Where, *Y*_ij_ = response variable for mineral block treatment,

*μ* = general average,

*α*_i_ = the effect of providing mineral blocks, and

*ε*_ij_ = random effects or error.

The data on mineral content, block physical properties, *in vitro* ruminal degradation, total gas production, and block palatability in response to treatment mineral blocks were statistically analyzed using analysis of variance (ANOVA). For the mineral block palatability data, the assumptions of normality and homogeneity of variances were verified using the Shapiro–Wilk and Levene’s tests, respectively. All assumptions were met prior to conducting ANOVA. When significant effects were detected, mean separation was performed using Duncan’s Multiple Range Test, provided by Co-Stat software [13].

## 3. Results

### 3.1. Mineral content and physical characteristics

The mineral content of Cu, Fe, and Mn in macroalgae *Gelidium* sp. was higher than the other three types of macroalgae (*Gracilaria* sp., *Sargassum* sp., and *Ulva* sp.) (*p* < 0.05) (Table 3). The mineral content of P in *Gelidium* sp. and *Gracilaria* sp. did not differ, but both were higher than in *Sargassum* sp. and *Ulva* sp. Similarly, the Zn concentration in *Gelidium* sp. and *Sargassum* sp. did not differ, but both were higher than in *Gracilaria* sp. and *Ulva* sp. The potassium (K) content in fortified MBFM blocks was higher than in the MBNF (*p* < 0.05) (Table 4). The mineral content of K, Cu, Fe, and Mn in MBFM-GE (*Gelidium* sp.) was higher than in the control MBNF (*p* < 0.05) (Table 4). The physical characteristics of MBFM, including height, diameter, volume, weight, and specific gravity, did not differ between blocks (Table 5). However, a difference was observed in the compressive strength of MBFM-GE, which was higher than MBNF (*p* < 0.05) (Table 5).

**Table 3.** Mineral content of macroalgae (*Gelidium* sp., *Gracilaria* sp., *Sargassum* sp., *Ulva* sp.).

Mineral content	Macroalgae			
	*Gelidium* sp.	*Gracilari*a sp.	*Sargassu*m sp.	*Ulva* sp.
	%			
Calcium (Ca)	12.2 ± 0.44^bc^	17.23 ± 2.01^a^	14.22 ± 2.81^ab^	9.03 ± 1.18^c^
Phosphorus (P)	0.34 ± 0.03^a^	0.30 ± 0.06^a^	0.22 ± 0.04^b^	0.13 ± 0.00^c^
Potassium (K)	2.45 ± 0.36^b^	6.44 ± 0.42^a^	6.60 ± 0.24^a^	2.40 ± 0.11^b^
Sodium (Na)	0.48 ± 0.07^b^	0.61 ± 0.01^a^	0.38 ± 0.02^c^	0.55 ± 0.02^ab^
Sulfur (S)	1.79 ± 0.23^b^	1.48 ± 0.27^b^	0.96 ± 0.02^c^	2.97 ± 0.05^a^
	mg/kg			
Copper (Cu)	66 ± 4^a^	37 ± 3^b^	44 ± 5^b^	30 ± 1^c^
Iron (Fe)	2800 ± 40^a^	2210 ± 33^b^	460 ± 17^d^	1240 ± 28^c^
Manganese (Mn)	870 ± 118^a^	309 ± 55^b^	291 ± 42^b^	326 ± 68^b^
Zinc (Zn)	120 ± 15^a^	75 ± 6^b^	136 ± 34^a^	43 ± 3^b^

Mineral content analysis by X-ray fluorescence (XRF) analysis; ^a, b, c, d,^ Different superscripts on the same rows indicate significant differences (*p* ˂ 0.05); Values are presented as mean ± standard deviation (SD).

**Table 4.** Mineral content of the mineral block is fortified by macroalgae and control (non-fortified).

Mineral content	MBFM				MBNF	*F*-value	*p*-value
	*Gelidium* sp.	*Gracilaria* sp.	*Sargassum* sp.	*Ulva* sp.			
	%						
Calcium (Ca)	19.76 ± 0.99	19.18 ± 1.14	19.68 ± 1.04	19.23 ± 1.9	18.46 ± 0.34	0.865	0.517
Phosphorus (P)	0.12 ± 0.03	0.11 ± 0.01	0.12 ± 0.03	0.12 ± 0.03	0.11 ± 0.01	0.097	0.981
Potassium (K)	0.21 ± 0.00^b^	0.28 ± 0.01^a^	0.21 ± 0.04^b^	0.22 ± 0.01^b^	0.18 ± 0.00^c^	11.609	0.001
Sodium (Na)	2.47 ± 0.41	2.98 ± 0.09	2.43 ± 0.19	2.41 ± 0.38	2.37 ± 0.07	2.590	0.101
Sulfur (S)	0.29 ± 0.04	0.29 ± 0.05	0.28 ± 0.02	0.32 ± 0.07	0.27 ± 0.11	0.275	0.887
	mg/kg						
Copper (Cu)	59 ± 3^a^	59 ± 2^a^	48 ± 2^b^	60 ± 3^a^	46 ± 1^b^	22.249	0.000
Iron (Fe)	3225 ± 305^a^	3000 ± 190^ab^	2640 ± 50^b^	2865 ± 235^ab^	2740 ± 40^b^	4.176	0.030
Manganese (Mn)	413 ± 37^a^	363 ± 6^b^	368 ± 11^b^	376 ± 26^b^	328 ± 12^c^	5.927	0.010
Zinc (Zn)	22 ± 2^b^	20 ± 1^b^	52. ± 4^a^	19 ± 2^b^	19 ± 2^b^	116.925	0.000

MBFM = mineral block fortified by macroalga; MBNF = mineral block non-fortified; ^a, b, c,^ Different superscripts on the same rows indicate significant differences (*p* ˂ 0.05); Values are presented as mean ± standard deviation (SD). Mineral content analysis by X-Ray Fluorescence.

**Table 5.** Physical characteristics of the mineral block fortified by macroalgae and control (non-fortified).

Parameters	MBFM				MBNF
	*Gelidium*> sp.	*Gracilaria* sp.	*Sargassum* sp.	*Ulva* sp.	
Height (cm)	11.87 ± 0.14	12.29 ± 0.14	12.06 ± 0.34	12.14 ± 0.21	12.56 ± 0.19
Diameter (cm)	8.68 ± 0.03	8.66 ± 0.04	8.60 ± 0.01	8.62 ± 0.03	8.41 ± 0.21
Volume (cm^3^)	702.50 ± 11.33	723.70 ± 13.50	699.99 ± 21.36	708.36 ± 13.58	697.99 ± 40.69
Weight (kg)	1.006 ± 0.09	1.03 ± 0.03	1.03 ± 0.012	1.02 ± 0.02	1.008 ± 0.06
Specific gravity (kg/dm^3^)	1.503 ± 128.31	1.421 ± 57.60	1.503 ± 128.31	1.433 ± 51.32	1.443 ± 49.70
Compressive strength (kg/cm^2^)	35.52 ± 9.79^a^	23.98 ± 2.69^b^	33.08 ± 2.20^a^	30.49 ± 7.17^a^	21.00 ± 5.72^b^

MBFM = mineral block fortified by macroalga; MBNF = mineral block non-fortified; ^a, b, c, d,^ Different superscripts on the same rows indicate significant differences (*p* ˂ 0.05); Values are presented as mean ± standard deviation (SD).

### 3.2. Ruminal degradability profiles and block palatability

**Figure 2.**
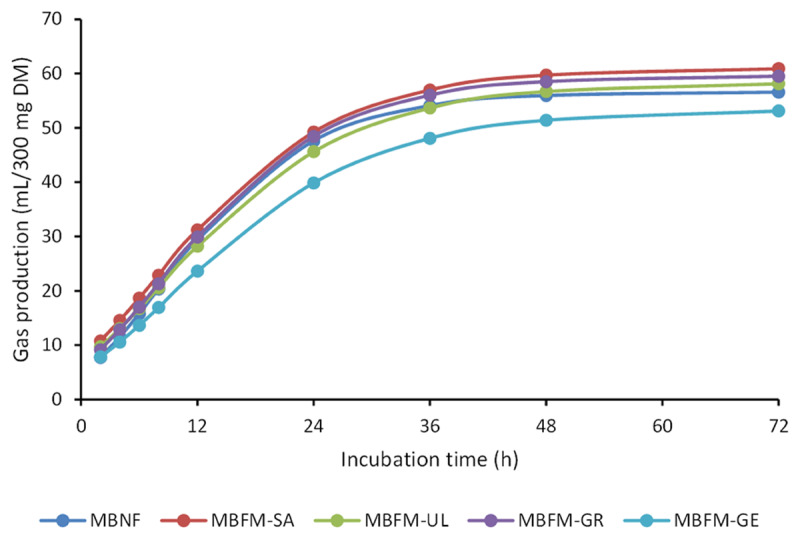
Gas production of a basal diet + mineral block fortified by macroalgae (MBFM-GE, MBFM-GR, MBFM-SA, MBFM-UL) and a basal diet + mineral block non-fortified (MBNF). MBFM-GE = mineral block fortified by *Gelidium* sp., MBFM-GR = mineral block fortified by *Gracilaria* sp., MBFM-SA = mineral block fortified by *Sargassum* sp., MBFM-UL = mineral block fortified by *Ulva* sp., MBNF = mineral block non-fortified.

**Figure 3.**
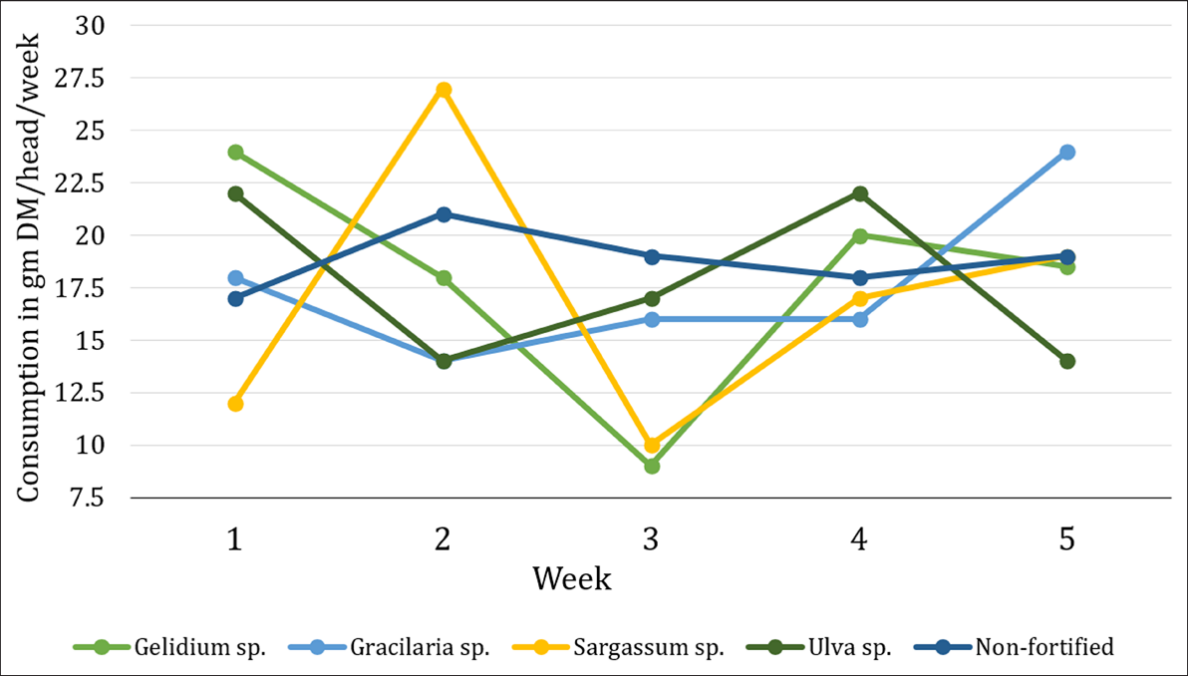
Weekly consumption of mineral blocks fortified with different macroalgae (*Gelidium* sp., *Gracilaria* sp., *Sargassum* sp., and *Ulva* sp.) and the non-fortified control over a 5-week period (mean values on a dry matter basis).

Fortification of the mineral block formula with all macroalgae increased *in vitro* DM digestibility (IVDMD) and *in vitro* organic matter digestibility (IVOMD) compared with the control non-fortified block (Table 6). The IVDMD and IVOMD of MBFM-GE (*Gelidium* sp.) resulted in the highest digestibility, exceeding the control values by 20.19% and 19.3% (*p* < 0.05), while for *Gracilaria* sp., *Sargassum* sp., and *Ulva* sp., the differences ranged from 6.59% to 17.68% in IVDMD and 5.61% to 16.21% in IVOMD, respectively. The gas production over 72 h of the five treatments is visualized in Figure 2, where fortified blocks have higher gas production than the control. The palatability of the MBFM (*Gelidium* sp., *Gracilaria* sp., *Sargassum* sp., and *Ulva* sp.) and MBNF blocks in Ongole crossbred bulls over a five-week period is presented in Figure 3.

**Table 6.** *In vitro* dry matter and organic matter digestibility of a basal diet + mineral block is fortified by macroalgae, and a basal diet + mineral block is non-fortified.

Parameters	A basal diet + MBFM				Basal diet + MBNF
	*Gelidium* sp.	*Gracilaria* sp.	*Sargassum* sp.	*Ulva* sp.	
IVDMD (%)	71.99 ± 11.43^a^	69.48 ± 14.30^ab^	58.39 ± 15.53^ab^	67.66 ± 7.47^ab^	51.80 ± 20.78^b^
IVOMD (%)	73.52 ± 15.55^a^	70.43 ± 14.03^ab^	59.83 ± 10.16^ab^	69.76 ± 9.38^ab^	54.22 ± 16.05^b^

IVDMD = *In vitro* dry matter digestibility; IVOMD = *In vitro* organic matter digestibility; A basal diet used *Pennisetum purpureum*; MBFM = mineral block fortified by macroalga; MBNF = mineral block non-fortified;^a, b,^ Different superscripts on the same rows indicate significant differences (*p* ˂ 0.05); Values are presented as mean ± standard deviation (SD).

## 4. Discussion

The use of non-fortified mineral blocks is common in Indonesian smallholder cattle systems [14, 15, 16], whereas the fortification of macroalgae has rarely been explored. Fortifying mineral blocks with tropical macroalgae, particularly *Gelidium* sp., significantly improves their nutritional composition and physical properties without reducing voluntary intake by Ongole crossbred bulls. The inclusion of 2% *Gelidium* sp. increased levels of K, Cu, Fe, and Mn compared with the non-fortified block, confirming the mineral richness of red macroalgae, as previously reported [17, 18, 19, 20]. The mineral content of macroalgae varies considerably across species, seasons, habitats, and processing technologies [21, 22, 23], which may explain the variations observed in this and other studies. X-ray fluorescence analysis confirmed that *Gelidium* sp. contained higher levels of Cu, Fe, and Mn than *Gracilaria* sp., *Sargassum* sp., or *Ulva* sp., which explains why MBFM-GE had the highest concentrations of these trace minerals among the treatments. Interestingly, Ca, P, Na, and S levels remained unchanged, suggesting that macroalgae fortification primarily enriches micro-mineral rather than macro-mineral content.

Technologically, macroalgae fortification improved the compressive strength of mineral blocks, with MBFM-GE showing a 69% increase compared to the non-fortified (control). This improvement can be attributed to the polysaccharides and gel-forming compounds naturally present in *Gelidium* sp., which act as effective binding agents [6, 24]. A stronger block is advantageous under field conditions because it resists breakage and reduces mineral losses caused by fragmentation or excessive licking, ultimately improving cost efficiency for smallholder farmers [25].

Ruminal fermentation studies revealed that the cumulative gas production from fortified and non-fortified blocks was similar, except for MBFM-SA, which produced significantly more gas at 6–36 h of incubation than MBFM-GE. Gas production is influenced by the fermentation pathways and the stoichiometry of volatile fatty acid formation [26], and differences in gas output do not always reflect true digestibility. This observation was supported by the *in vitro* digestibility results, which showed that Gelidium-fortified blocks significantly increased IVDMD (by 39%) and IVOMD (by 36%) compared to the control, despite producing slightly less gas. Such findings are consistent with the notion that gas production techniques may not linearly predict digestibility [27]. The improved digestibility is likely associated with the elevated Mn and Fe contents of MBFM-GE, as Mn has been shown to stimulate amylase and trypsin activities and enhance rumen microbial growth [28, 29]. Alternatively, bioactive compounds in *Gelidium* sp., such as soluble polysaccharides, could have enhanced microbial activity and fermentation efficiency [30, 31].

Palatability was unaffected by fortification, with an average intake of 93.11 gm DM/head/week across treatments. This suggests that including macroalgae at 2% does not deter consumption, which is crucial because any nutritional improvement must not reduce voluntary intake. Our results align with previous reports that moderate levels of macroalgae (≤ 20% of diet DM or 90 gm/kg) are well accepted by ruminants [32], though certain species or higher inclusion rates can decrease palatability [33, 34, 35, 36]. Maintaining intake is critical to ensure that the benefits of improved mineral profiles and digestibility translate into better animal performance.

Overall, these findings confirm that tropical macroalgae, especially *Gelidium* sp., are promising natural fortifiers for mineral blocks. They enhance trace mineral enrichment, improve the compressive strength of the block, and increase *in vitro* digestibility without compromising palatability. These results are consistent with recent research indicating that macroalgae-based feed additives can enhance the nutrient efficiency and sustainability of ruminant production systems [19, 37, 38]. Future research should focus on dose–response studies, *in vivo* digestibility trials, growth performance evaluations, and characterization of the bioactive compounds responsible for these beneficial effects.

## 5. Conclusions

Fortifying mineral blocks with *Gelidium* sp. improved trace mineral content, compressive strength, and *in vitro* digestibility without reducing palatability, confirming its potential as an effective mineral supplementation strategy for Ongole crossbred bulls. These findings confirm the potential of tropical macroalgae as a natural fortifier and provide a basis for further *in vivo* studies to optimize mineral supplementation strategies.

## Data Availability

The data presented in this study are available from the corresponding author upon reasonable request.

## List of abbreviations

MBNF, mineral block non-fortified; MBFM, mineral block fortified with macroalga; MBFM-GE, mineral block fortified with *Gelidium* sp.; MBFM-GR, mineral block fortified with *Gracilaria* sp.; MBFM-SA, mineral block fortified with *Sargassum* sp.; MBFM-UL, mineral block fortified with *Ulva* sp.; DM, dry matter; OM, organic matter; IVDMD, in vitro dry matter digestibility; IVOMD, *in vitro* organic matter digestibility; BW, body weight; h, hours; cm, centimeters; m, meter; ml, milliliter; kg, kilograms; mm, millimeter.
